# Optimized Analysis of Ergot Alkaloids in Rye Products by Liquid Chromatography-Fluorescence Detection Applying Lysergic Acid Diethylamide as an Internal Standard

**DOI:** 10.3390/toxins11040184

**Published:** 2019-03-28

**Authors:** Iris Holderied, Michael Rychlik, Paul W. Elsinghorst

**Affiliations:** 1 Central Institute of the Bundeswehr Medical Service Munich, Ingolstädter Landstraße 102, D-85748 Garching, Germany; 2 Chair of Analytical Food Chemistry, Technical University of Munich, Maximus-von-Imhof-Forum 2, D-85350 Freising, Germany; 3 Pharmaceutical Institute, Pharmaceutical Chemistry I, University of Bonn, An der Immenburg 4, D-53121 Bonn, Germany

**Keywords:** ergot alkaloids, rye products, LSD, LC-FLD

## Abstract

Analysis of ergot alkaloids remains a topic of importance and the European Food Safety Authority (EFSA) has encouraged laboratories to provide monitoring data for the further evaluation of their occurrence in food and feed. While LC-MS/MS has dominated developments in recent years, LC-FLD is still more widespread, especially in developing countries. To improve the analysis of ergot alkaloids by LC-FLD, we developed an improved protocol introducing lysergic acid diethylamide (LSD) for internal standardization. Several aspects such as the composition and pH of the extraction medium, type of sorbent and conditions applied for solid-phase extraction/clean-up, use of a keeper during final evaporation and the type of syringe filter used for filtration prior to injection were thoroughly investigated. Optimized conditions comprise extraction by ethyl acetate, methanol and 28% aqueous ammonia in combination with basic aluminum oxide for extract clean-up. Use of a keeper was found inappropriate as LC-FLD analysis was significantly affected by co-eluting keeper components. Similar observations were made with some of the investigated syringe filters, where polytetrafluoroethylene (PTFE) proved to be the most suitable. Validation and application of the optimized methodology to real samples provided limits of detection and quantification suitable for the evaluation of relevant ergot alkaloid contaminations in rye and bakery products with superior precision that was facilitated by the introduced internal standard, LSD.

## 1. Introduction

Rye grain, flour and bakery products are well known for their possible contamination by ergot alkaloids, because of the prominent sclerotia formed by *Claviceps purpurea* when it grows on rye. However, other cereals such as wheat, barley, millets and oats can also be affected, sometimes by different species like *Claviceps africana* producing a slightly modified ergot alkaloid spectrum [1]. From the many alkaloids produced by the genus *Claviceps,* a representative set of 12 predominant ergot alkaloids (ergometrine, ergotamine, ergosine, ergocornine, ergokryptine, ergocristine and their corresponding inine-epimers) has been identified, which has been the subject of a detailed evaluation by the European Food Safety Authority (EFSA) [2]. Based on toxicological studies of their vasoconstrictive effects and following an estimate of human and animal dietary exposure by the EFSA, a group tolerable daily intake of 0.6 µg/kg bodyweight has been derived [3]. To monitor human and livestock exposure and to support minimization strategies for crop production, ergot alkaloids are subject to routine analysis throughout Europe [4].

Quantitative analysis of ergot alkaloids is mostly achieved by liquid chromatography, using either fluorescence or tandem mass spectrometry for detection [5]. Although LC-MS/MS has become the method of choice in recent years [6,7,8], LC-FLD still provides a reliable alternative as it is sensitive enough to quantify toxicologically relevant levels. For either technique, a significant impact on overall analytical performance originates from sample preparation, mainly from necessary extraction and clean-up steps. High recoveries are usually achieved when using a two-phasic extraction mixture of ethyl acetate, methanol and aqueous ammonia followed by filtration through basic alumina [9]. As extraction pH shows a considerable influence on epimerization, e.g., the ratio of ergometrine to ergometrinine, the analysis preferably addresses each epimer pair in total. Further improvement with respect to accuracy and precision can be achieved through the application of an internal standard. As such, methysergide and dihydro-ergocristine have been used for LC-MS/MS analysis of food [5,10], but no reports exist for methods based on LC-FLD.

Here we report on a robust and reliable procedure for the analysis of ergot alkaloids by LC-FLD, using the structurally related but non-natural lysergic acid diethylamide (LSD) as an internal standard. This method has been successfully applied to rye flour and respective bakery products such as bread, buns and crispbread.

## 2. Results and Discussion

With the objective to establish a straightforward and reliable LC-FLD method for the quantification of the 12 representative ergot alkaloids in rye products, a special focus was put on the optimization of reported protocols for sample preparation in addition to the implementation of a suitable internal standard. Originating from the extraction procedure described by Müller et al. [9], several parameters were thoroughly evaluated for their optimization potential: (1) composition and pH of the extraction medium, (2) type of sorbent and conditions applied for the solid-phase extraction/clean-up, (3) use of a keeper during final evaporation and (4) type of syringe filter used for filtration prior to injection.

**Figure 1:**
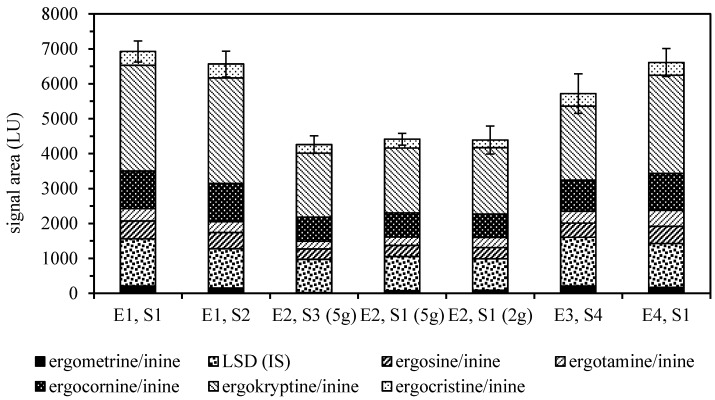
Absolute recoveries of ergot alkaloids and the lysergic acid diethylamide (LSD) internal standard (IS) from rye flour spiked with ground sclerotia compared by LC-FLD signal areas (± SD, *n* = 6). Extraction solvents: E1, ethyl acetate/methanol/28% aqueous ammonia, 75/5/7 *v*/*v*; E2, dichloromethane/ethyl acetate/methanol/28% aqueous ammonia, 50/25/5/1 *v*/*v*; E3, acetonitrile/0.02% aqueous ammonium carbonate 84/16 *v*/*v*; E4, methanol/0.013 M aqueous phosphoric acid 7/3 *v*/*v*. Clean-up sorbents: S1, basic aluminum oxide; S2, acidic aluminum oxide; S3, neutral aluminum oxide; S4, MycoSep^®^ 150 Ergot.

Various solvent mixtures of different pH have been reported for the extraction of ergot alkaloids, but none of them appear to show optimum properties [5]. Two-phasic systems, such as mixtures of ethyl acetate or dichloromethane with an aqueous phase often containing ammonia, have been used for many years. Recent reports usually favor polar extraction solvents like acetonitrile or methanol in combination with buffer systems of either basic or acidic pH, as they facilitate subsequent analysis by LC-MS/MS. A similar situation can be seen with the typical clean-up most often applied to primary extracts, where a variety of sorbents have been reported. They usually provide recovery ratios above 80%, except for the more polar ergometrine/ergometrinine pair that typically shows a lower recovery. In our tests, ethyl acetate, methanol and 28% aqueous ammonia in combination with basic aluminum oxide provided the best overall recovery results (Figure 1).

**Figure 2:**
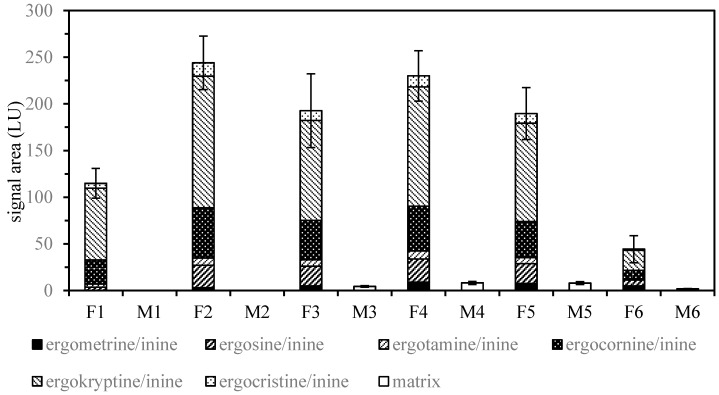
Ergot alkaloid (F1–F6) and matrix (M1–M6) signals of fractions 1–6 (1 mL) from solid-phase extraction/clean-up using a combination of ethyl acetate/methanol/28% aqueous ammonia (75/5/7 *v*/*v*) and basic aluminum oxide to extract rye flour spiked with ground sclerotia (± SD, *n* = 6). Fractions 4 to 6 were obtained by additional flushing of the aluminum oxide cartridges. Fraction 4 and 5 show remarkable levels of ergot alkaloids and were therefore included for subsequent LC-FLD analysis.

Solid-phase extraction/clean-up of mixtures covering a wide range of polarity generally leads to a compromise between analyte recovery and matrix removal based on the volumes applied for washing and elution. Using a polar-retaining sorbent like aluminum oxide, more polar components, e.g., ergometrine/ergometrinine, may be discriminated with small elution volumes. As shown in Figure 2, additional fractions (4–5 mL) obtained by flushing the aluminum oxide cartridges still contained remarkable amounts of ergot alkaloids when compared to the standard pass-through protocol (3 mL) [9]. Further investigation of the total extraction volume subjected to solid-phase extraction/clean-up provided conditions for increased recovery with reasonable matrix elution.

Ergot alkaloids are known for a tendency to epimerize, as well as for their susceptibility to degrade as a result of light or heat exposure. Aprotic solvents have been reported to stabilize ergot alkaloids in solution [11], but it appears that recommended storage solvents such as chloroform fail to completely re-dissolve ergot alkaloid lyophilisates (the preferred method of commercial supply). To evaluate a similar impact on the reconstitution of evaporated extracts for LC-FLD analysis, recovery rates in the absence and presence of a keeper were compared. At first, ethylene glycol (100 µL) as well as glycerol (100 µL) seemed to provide highly improved recovery rates for ergometrine/ergometrinine, but on careful inspection it was discovered that the co-eluting keeper induced only an increased fluorescence of these polar analytes rather than a real increased recovery. Therefore and as 20% aqueous phosphoric acid (25 µL) caused dramatic analyte losses, the idea of adding a keeper to improve recovery was dropped.

Moreover, an interesting observation was made when we explored other possible interferences originating from the reconstitution process. Comparison of the syringe filters used for final filtration revealed that polyvinylidene difluoride (PVDF)- and polyethylene terephthalate (PET)-derived filters leaked one or more substances that interfered with fluorescence detection. As this was not observed for either polytetrafluoroethylene (PTFE)- or regenerated cellulose (RC)-derived filters, these two can be recommended with respect to ergot alkaloid analysis and possibly other LC-FLD methods.

To further improve LC-FLD analysis, the introduction of an internal standard was subsequently investigated as it had already been applied to the LC-MS/MS analysis of ergot alkaloids. As lower recovery rates were primarily an issue for the more polar ergot alkaloids, especially ergometrine/ergometrinine and ergosine, an ergot analogue of similar polarity and elution behavior was considered optimal. Searching for a structurally related but non-natural candidate, we selected lysergic acid diethylamide (LSD), as it is sufficiently stable and commercially available.

**Figure 3:**
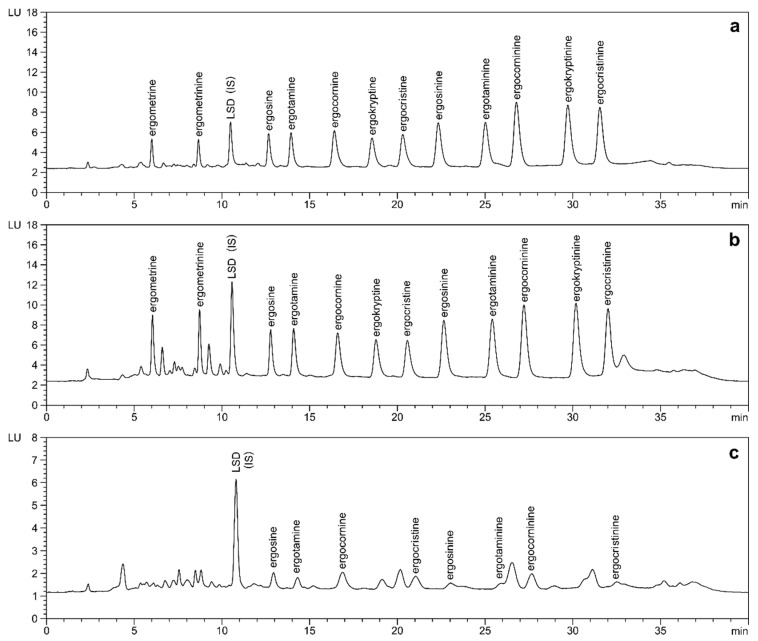
LC-FLD analysis of ergot alkaloids in rye flour (**a**) and buns (**b**) spiked to 50 µg/kg of each ergot alkaloid and LSD (internal standard, IS) in comparison to naturally contaminated rye flour (**c**); ergosine: 11.3 µg/kg, ergotamine: 10.9 µg/kg, ergocornine: 15.3 µg/kg, ergocristine: 9.2 µg/kg, ergosinine: 4.3 µg/kg, ergotaminine: 3.1 µg/kg, ergocorninine: 8.5 µg/kg, ergocristinine: 3.9 µg/kg. Separation after optimized extraction and clean-up was achieved on a phenyl-hexyl column (4.6 × 100 mm, 3.0 µm) with gradient elution using 0.02% aqueous ammonium bicarbonate/acetonitrile.

As shown in Figure 3, LSD was found to elute within the polar region between ergometrinine and ergosine, which in bakery products such as bread and buns usually shows some co-eluting matrix interferences. Except for ergometrine/ergometrinine (recovery 61%, precision 10.5%), both overall recovery (96–116%) and precision (1.6–4.5%) were significantly improved by the application of the internal standard (Table 1). The persistent difficulties with ergometrine/ergometrinine can be attributed to incomplete extraction or clean-up, as comparable results were obtained when detection was carried out by MS/MS (data not shown). Nevertheless, any physical losses during sample preparation will be compensated for by the application of the internal standard.

Table 1 summarizes the results of the subsequent validation of the optimized methodology when applied to real samples such as bakery products. The derived limit of detection (LOD) and limit of quantification (LOQ) levels are well in line with comparable literature reports that apply LC-FLD, which also holds true for the recovery rates obtained [9,12,13]. The only exceptions are ergometrine/ergometrinine, which showed slightly higher LOD/LOQ values with lower recovery rates. However, as the determination of these values depends substantially on the methodology applied (signal to noise, blank or calibration method as in this study), careful analysis of the reported values is a prerequisite for appropriate comparison. The absolute levels of quantification achieved in the low µg/kg range are sufficient to evaluate relevant contaminations of rye and bakery products. If required, significantly lower LOD and LOQ levels may be achieved by MS/MS detection, which in our tests ranged in the lower ng/kg range (data not shown). For most of the analyzed ergot alkaloids and rye products CVs at a level of 50 µg/kg were below or close to 5%, which is very satisfying for a precision achieved by applying an internal standard.

## 3. Conclusions

In summary, the reported method successfully applies LSD for the first time as an internal standard for ergot alkaloid quantification by LC-FLD in rye flour and rye products such as bread, buns and crispbread. It provides valuable improvements for laboratories that are considering ergot alkaloid analysis but do not own an LC-MS/MS system, or whenever other circumstances advise the use of LC-FLD. The evaluation of further optimization options such as a simple freeze-out clean-up, which has recently been reported by Schummer et al. [14], is currently underway in our laboratories.

## 4. Materials and Methods

### 4.1. Chemicals and Reagents

All reference materials (ergometrin/-inine, ergosin-/inine, ergotamine/-inine, ergocornin/inine, ergocristin/-inine, α-ergokryptin/-inine) were obtained from Romer Labs (Butzbach, Germany), while the LSD used as the internal standard was from LGC (Wesel, Germany). Acetonitrile (LC-grade), ethyl acetate and methanol were purchased from VWR (Darmstadt, Germany), while the aqueous ammonia (28%) was from Merck (Darmstadt, Germany) and the ammonium bicarbonate (LC-grade) was from Fluka (Seelze, Germany).

### 4.2. Samples

The ergot sclerotia that were used for method development were kindly provided by the Bavarian Millers Association (Munich, Germany) and Marie-Luise Koch (University of Bonn, Bonn, Germany). Flour and bakery products were obtained from local suppliers. All samples were carefully dried, finely ground and stored at room temperature prior to analysis.

### 4.3. Stock Solutions

Stock solutions of the ergot alkaloids and the internal standard (LSD) were prepared by dissolving and diluting the commercial lyophilisates (ergot alkaloids) or solids (LSD) in acetonitrile to a final concentration of 1 µg/mL (the use of chloroform as suggested by Hafner et al. [11] did not allow for complete dissolution). Mixed calibrants containing 10 ng/mL LSD and all analytes at concentrations of 5, 10, 25, 50 and 100 ng/mL were subsequently prepared by dilution with 0.02% aqueous ammonium bicarbonate and acetonitrile (50/50 *v*/*v*).

### 4.4. Sample Preparation

For extraction, each sample (10 g) was placed in a 250 mL PTFE centrifugation bottle, spiked with LSD (1 µg/mL, 500 µL) and ethyl acetate/methanol/28% aqueous ammonia (75/5/7 *v*/*v*, two-phasic mixture that must be thoroughly shaken prior to use; 50 mL) was added. The mixture was shaken (250 rpm, 45 min), then centrifuged (3.000× *g*, 10 °C, 20 min) and 4 mL was transferred to an aluminum oxide cartridge (Sep-Pak^®^ Alumina B 12 cc Vac cartridge, 2 g, 50–300 µm; Waters, Eschborn, Germany). Following complete filtration, the cartridge was flushed by the addition of ethyl acetate/methanol/28% aqueous ammonia (75/5/7 *v*/*v*, 1 mL), then the combined filtrates were thoroughly mixed and 1 mL was evaporated using a vacuum concentrator (Christ, Osterode, Germany). Reconstitution using 0.02% aqueous ammonium bicarbonate and acetonitrile (50/50 *v*/*v*, 1 mL), sonication for 10 min and filtration using a PTFE syringe filter provided the final sample solution (amber glass vials are recommended for light protection).

### 4.5. LC-FLD Conditions

Chromatography was carried out using a 1200 LC system from Agilent (Waldbronn, Germany) that was equipped with a fluorescence detector (λ_ex_: 245 nm, λ_em_: 430 nm, PMT gain: 14). Chromatographic separation was achieved on a Phenomonex Luna phenyl-hexyl column (4.6 × 100 mm, 3.0 µm particle size; Phenomenex, Aschaffenburg, Germany) applying gradient elution (0.02% aqueous ammonium bicarbonate/acetonitrile, 80/20 (0 min, 0.6 mL/min), 70/30 (1 min, 0.6 mL/min), 50/50 (9 min, 0.6 mL/min), 45/55 (15 min, 0.3 mL/min), 40/60 (22 min, 0.3 mL/min), 30/70 (30 min, 0.3 mL/min), 20/80 (33 min, 0.3 mL/min), 80/20 (36 min, 0.6 mL/min), 80/20 (40 min, 0.6 mL/min), 30 °C) with an injection volume of 20 µL. Samples were stored in the autosampler at 5 °C.

### 4.6. Method Validation

Linearity was initially assessed using an eluent-based, five-point calibration (5, 10, 25, 50 and 100 ng/mL of each ergot alkaloid) with LSD as the internal standard (10 ng/mL). Limits of detection (LOD, S/N = 3) and quantification (LOQ, S/N = 9) were subsequently derived by applying a matrix-matched approach using spiked rye flour. Precision and recovery data were obtained from three- to six-fold analysis of spiked rye flour, bread and bun samples at 50 µg/kg of each ergot alkaloid, to reflect an estimated ergot alkaloid content of 60% of the maximum allowed level for sclerotia in rye grain (0.05% sclerotia containing 0.2% ergot alkaloids).

## Figures and Tables

**Table 1:** Validation data from three- (bread, buns and crispbread) and six-fold (flour) analysis of ergot alkaloids in spiked rye products using LSD as the internal standard (recovery and precision (CV) at 50 µg/kg as sums of the corresponding epimers). LOD: limit of detection, LOQ: limit of quantification.

Ergot Alkaloid	Flour				Bread		Whole-Grain Bread		Buns		Crispbread	
	LOD	LOQ	Recovery	CV	Recovery	CV	Recovery	CV	Recovery	CV	Recovery	CV
	(µg/kg)		(%)		(%)		(%)		(%)		(%)	
ergometrine	14.9	44.6	60.9	10.5	66.8	16.7	46.3	5.2	62.8	5.7	67.9	8.1
ergometrinine	12.8	38.4										
ergotamine	4.8	14.5	96.1	1.6	87.7	1.6	84.0	5.2	101.7	1.6	99.5	5.2
ergotaminine	3.3	10.0										
ergosine	9.0	27.1	97.8	4.5	96.4	3.3	89.3	6.0	103.0	1.3	116.5	2.6
ergosinine	3.3	10.0										
ergocornine	4.7	14.2	108.3	2.5	95.4	3.1	91.3	6.2	108.1	1.4	106.4	18.6
ergocorninine	1.4	4.1										
ergokryptine	5.8	17.4	102.2	4.4	100.1	2.4	98.4	5.7	111.9	1.6	117.5	12.8
ergokryptinine	1.4	4.3										
ergocristine	4.7	14.2	115.6	3.9	102.0	3.3	94.9	6.3	116.8	2.9	106.7	3.9
ergocristinine	1.5	4.6										
